# “Appropriateness and adequacy of antibiotic prescription for upper respiratory tract infections in ambulatory health care centers in Ecuador”

**DOI:** 10.1186/s40360-018-0237-y

**Published:** 2018-07-27

**Authors:** Xavier Sánchez Choez, María Luciana Armijos Acurio, Ruth E. Jimbo Sotomayor

**Affiliations:** 1 Pontificia Universidad Católica del Ecuador, Posgrado de Medicina Familiar, Avenida 12 de Octubre 1076, Vicente Ramón Roca, Quito, Ecuador; 2 Universidad Alcalá de Henares, Alcalá de Henares, Spain

**Keywords:** Antibiotic, Prescription, Family practice, Health systems and services, Anti-bacterial agents, Drug utilization, Practice guidelines

## Abstract

**Background:**

Upper respiratory tract infections are the leading cause of misuse of antibiotics, a problem that leads to unnecessary adverse events and antibiotic resistance. Antibiotic prescription in Ecuador was analyzed in order to evaluate the state of antibiotic prescribing for upper respiratory tract infections. Both the appropriateness and adequacy of prescribing was evaluated. Appropriateness represents the percentage of prescriptions that are indicated; adequacy refers to the percentage of patients requiring antibiotics who are treated.

**Methods:**

The aim of the study is to analyze the appropriateness and adequacy of antibiotic prescription for upper respiratory tract infections in the Ambulatory Health Centers of the Ministry of Public Health of Ecuador. This is a cross-sectional study of patients from one Health Center of the Ministry of Public Health in the District 17D03 in Ecuador during 2015 with upper respiratory tract infection as a primary diagnosis.

**Results:**

We included a total of 1393 patients in the analysis. Out of the 1393 patients identified, 523 were prescribed antibiotics, constituting an antibiotic prescription rate of 37.5%, and 51 required antibiotics, reflecting a real need of antibiotics of 3.7%. Appropriateness: Of these 523 patients who were treated, 51 required an antibiotic, resulting in an appropriate antibiotic prescription rate of 9.75%. Adequacy: When analyzing each individual case, 33 of these 51 patients received an antibiotic, constituting an adequate prescription rate of 64.7%.

**Conclusions:**

The results of our study report a 90.25% of inappropriate prescription. The antibiotic prescription, appropriate prescription, and adequate prescription rates show the need for implementation of strategies in order to reduce them. Related aspects regarding prescriber’s behavior and the patient’s expectations should be analyzed.

**Electronic supplementary material:**

The online version of this article (10.1186/s40360-018-0237-y) contains supplementary material, which is available to authorized users.

## Background

Appropriate and adequate use of drugs is now one of the most commonly debated topics in public health. According to the WHO, only 50% of all prescribed medications are dispensed or sold in an appropriate way [1]. On the other hand, about one third of the world population lacks access to drugs considered as essential drugs by the WHO [2]. According to the WHO, the rational use of drugs requires that “patients receive medications appropriate to their clinical needs, in doses that meet their own individual requirements, for an adequate period of time, and at the lowest cost to them and their community” [3]. The appropriate prescription rate is the number of cases that need treatment divided by the number of cases that receive it. It has the objective of maximizing the effectiveness of a treatment, minimizing the risk and costs, and taking into consideration the preference of the patients [4]. Adequate prescription rate is the number of cases that needs and receives treatment divided by the number of cases that need it. Adequate prescription allows us to analyze if patients that require treatment are receiving it.

Differences in prescription can be due to both the prescriber and the patient. Studies have shown that factors like gender, multi-morbidity and previous personal experiences can influence prescription [5, 6]. Prescription is also influenced by the health provider’s knowledge of evidence based medicine, current guidelines, personal experience and lack of time or availability of drugs [7].

A 2016 study by Fleming-Dutra et al. in the United States shows a rate of inappropriate oral antibiotic prescription of about 50% in upper respiratory tract infection (URTI), adjusted by age and diagnostic in adults and children [8]. Another study in children in France finds results that suggest a 76% rate of inappropriate prescription of antibiotics for any pediatric diagnosis in primary health care and that URTI are the leading cause of misuse of antibiotics. [9].

A prospective study with 3402 patients with acute cough from 13 European countries analyzed the variation in prescription of antibiotics in primary care. Their results showed a high prescription rate, in all countries included that ranged between 20 and 90% (media of 53%) [10]. Another study from four Latin American countries found that 33% of patients with a suspicion of URTI in primary care centers that were included in the study were prescribed antibiotics [11].

Even though resistance to antibiotics is a natural phenomenon in bacteria, human activities have potentiated its expansion, favoring the appearance of resistant strains. The lack of efficient practices for prevention and control of infections has also contributed to the emergence of new resistances [12]. The inappropriate use of antimicrobials increases the risk of drug-resistant microorganisms [13].

Costelloe, et al. in 2010, demonstrate a strong association between the prescription of antibiotics in primary care and antimicrobial resistance in URTI [14]. It has been reported that antibiotic resistance to microorganisms such as *Staphylococcus aureus*, enterococci, and gram-negative bacilli, are associated with an increase in mortality, morbidity, days of hospitalization and health costs [15]. The increase in health costs associated to antibiotic resistance is due to the longer duration of disease and the need for additional diagnostic tests and high-cost antibiotics. [16].

Due to the large impact on health, antibiotic prescription in Ecuador was analyzed in order to evaluate if the tendency aligns with the misuse of antibiotics occurring around the world. Defining the scope of the problem will allow for further analysis and policy implementation in the country.

## Methods

The aim of this study was to analyze the appropriateness and adequacy of antibiotic prescription for URTI in the Ambulatory Health Centers of the Ministry of Public Health of Ecuador in the District 17D03 during 2015. The design of this cross-sectional study of the appropriateness and adequacy of antibiotic prescription imitates similar studies from different settings [8, 17–19] allowing us to obtain comparable results. We considered “Appropriate prescription rate” as the number of appropriate antibiotic prescriptions divided by all patients receiving antibiotics. Our definition of “Adequate prescription rate” is the number of patients that need and receive antibiotics divided by the number of patients that need it. The purpose of measuring the appropriate prescription rate is to identify the over-prescription that could be occurring. On the other hand, the purpose of analyzing the “Adequate prescription rate” is to identify cases of patients that needed antibiotics and did not receive treatment.

### Data source

The data source for the variables included in this study are the Electronic Health Records (EHR) of all the patients from one Health Center of the Ministry of Public Health in the District 17D03 in Ecuador during 2015 with URTI as a primary diagnosis. This district has been using EHR since 2010. Prescribers enter the information directly into the EHR on a computer during the outpatient appointment. Information was manually extracted from the EHR simultaneously by two peer reviewers, according to the following criteria:

*Inclusion criteria:* Patients 3 months of age and above who required clinical care for URTI in the Ambulatory Health Centers of the Ministry of Public Health in the District 17D03 in 2015.

*Exclusion criteria: 1)* Patients who received clinical care in the ambulatory health centers of the Ministry of Public Health that are not a part of the D1703 district; 2) Patients who received clinical care in ambulatory health centers that are not a part of the Ministry of Public Health in the D1703 district.

The International Classification of Disease (ICD) – 10 was used for selection of the diagnosis criteria for URTI, where we considered codes J00-J06, J10, J11, H65, H66 (Table 1). After individual data extraction, information was compared, and reached a consensus of inclusion or exclusion for each patient. An additional text file of description of variables shows the variables collected for the analysis of appropriateness and adequacy of antibiotic prescription (see Additional file 1).

**Table 1:** ICD-10 Codes considered as “upper respiratory tract infections” for the purpose of this study

We considered an upper respiratory tract infection diagnosis as any diagnosis registered according to the ICD-10 classification, as any the following: ᅟ• JOO Acute nasopharyngitis [common cold] ᅟ• JO1 Acute sinusitis (includes J01.0, J01.1, J01.2, J01.3, J01.4, J01.8, J01.9) ᅟ• JO2 Acute pharyngitis (includes J02.0, J02.8, J02.9) ᅟ• JO3 Acute tonsillitis (includes J03.0, J03.8, J03.9) ᅟ• JO4 Acute laryngitis and tracheitis (includes J04.0, J04.1, J04.2) ᅟ• JO5 Acute obstructive laryngitis [croup] and epiglottitis ᅟ• JO6 Acute upper respiratory infections of multiple and unspecified sites (includes J06.0, J06.8, J06.9) ᅟ• J10 Influenza due to other identified influenza virus (includes J10.1) ᅟ• J11 Influenza due to unidentified influenza virus (includes J11.1) We will consider as part of the upper respiratory tract infections, diseases of the middle ear categorized according to ICD-10 as: ᅟ• H65 Nonsuppurative otitis media ᅟ• H66 Suppurative and nonspecified otitis media	

The patients included in this study were those attended by the 5 types of health professionals that work in the designated health center and that diagnosed patients with URTI. These health professionals are classified as:

- Rural Medical Trainees: Medical Doctor that has recently completed their degree and is performing a year of community service as a health professional in a rural location as a prerequisite for medical practice in Ecuador.
- General Practitioner: Medical Doctor that has completed the year of community service as a health professional and can practice medicine freely in Ecuador.
- Family Medicine Doctor: Medical Doctor that has performed a postgraduate degree of 3 years in General Practice and is considered a specialist in Ecuador.
- Psychiatrists: Medical Doctor that has performed a postgraduate degree of 3 years in Psychiatry and is considered a specialist in Ecuador.
- Pediatricians: Medical Doctor that has performed a postgraduate degree of 3–4 years in Pediatrics and is considered a specialist in Ecuador.

### Sample

The sample was defined as all the patients that met the inclusion criteria and that had complete information in their EHR. Our sample is of 1393 patients.

#### Assessment of appropriateness and adequacy of antibiotic prescription

For this study, “Appropriate antibiotic prescription” is considered according to the standards defined in the Clinical Guideline for “Respiratory Tract Infections (self-limiting): prescribing antibiotics CG69” by the National Institute for Health and Care Excellence (NICE), considering the lack of a National clinical guideline for URTI in Ecuador. Table 2 describes the criteria for cases that needed antibiotic prescription according to each ICD category, as described in the NICE Clinical Guideline. Need for antibiotics was assessed by two reviewers separately in order to identify the cases that met the criteria for antibiotic prescription. When a data inconsistency was found in the EHR, the agreement was reached by consensus by the reviewers. We assessed appropriateness of the prescription by identifying the cases that needed treatment divided by the number of cases that received it. We assessed adequacy of the prescription by identifying individual patients that required antibiotic treatment according to Table 2 and evaluated if they received it or not.

**Table 2:** Guide for Classifying Appropriate Antibiotic Prescription: using ICD-10 codes to classify appropriate diagnosis of disease according to the symptoms and signs presented in the patients in the sample

ICD-10 code	Criteria for Appropriate Antibiotic Prescription
H65 Nonsuppurative otitis media H66 Suppurative and nonspecified otitis media	H65 must meet both conditions: presence of Acute Bilateral Otitis media AND age < 2 H65 or H66 with presence of otorrhea
J01 Acute sinusitis	Must meet all conditions: Fever of >38C°, purulent discharge and facial pain
JO2 Acute pharyngitis JO3 Acute tonsillitis	Must meet 3 of the following CENTOR criteria: ­ Presence of tonsilar exudate ­ Presence of painful anterior cervical lymphadenopathy or lymphadenitis ­ Fever (> 38°) ­ Absence of cough
JOO Acute nasopharyngitis [common cold] JO1 Acute sinusitis JO2 Acute pharyngitis JO3 Acute tonsillitis JO4 Acute laryngitis and tracheitis JO5 Acute obstructive laryngitis [croup] and epiglotitis JO6 Acute upper respiratory infections of multiple and unspecified sites J10 Influenza due to other identified influenza virus J11 Influenza due to unidentified influenza virus	Must meet any of the following criteria: ­ Presence of one or more of the following comorbidities: Cardiac, Pulmonary, Renal, Hepatic, Neuromuscular, Immunosuppression, Cystic Fibrosis, Diabetes Mellitus. - OR ­ Age < 2 years old AND history of prematurity OR ­ Age > 65 years old AND presence of cough AND two or more of the following: ▪ Hospitalized in 2014 ▪ Diabetes mellitus ▪ History of cardiac arrest ▪ Current use of corticosteroids OR ­ Age > 80 years old AND presence of cough AND one or more of the following: ▪ Hospitalized in 2014 ▪ Diabetes mellitus ▪ History of cardiac arrest ▪ Current use of corticosteroids

There are no ICD-10 codes for J07 and J08. J09 excluded because it refers to influenza and pneumonia

The antibiotic prescription rate was defined as the number of antibiotic prescriptions divided by all patients diagnosed with URTI.

### Statistical analysis

The variables included in this study were qualitative dichotomous and categorical variables and quantitative continuous variables, an additional text file of description of variables shows this in more detail (see Additional file 1). We performed a descriptive analysis with qualitative variables through frequency distributions, proportions and rates; and of quantitative variables through measures of central tendency and dispersion. We then performed statistical tests to determine the association between antibiotic prescription and several variables through a logistic regression. This regression analyzes the relationship between a dependent variable, antibiotic prescription, and independent variables, controlling for potential confounders. The independent variables were chosen by theoretical relationship to the dependent variable and through bivariate logistic regression models, an additional text file of variables considered for the regression models shows this in more detail (see Additional file 2). In the model we only included variables with a *p* value of < 0.25. These variables were: gender of prescriber, hours dedicated to clinical practice and category of health professionals. We excluded patient age, prescriber age and gender of patient due to their lack of statistical significance.

For the only categorical variable included, category of health professionals, we analyzed each category and decided to exclude Psychiatrists, due to the low number of consults included in the data. Then we reintroduced some variables that according to literature could be potential confounders (prescriber age and patient age) one by one creating an extended model, but did not find anything of significance. In order to choose between the models to be used, we used Akaike’s Information Criteria (AIC). The formula for the final model was:

### Log(antibiotic prescription) = b0 + b1*i.levelhp_cate + b2*gender_prescriber + b3*hours_clinical

Where *i.levlehp_cate* is health professional categorized by type, *gender_prescriber* is the gender of the prescriber, and *hours_clinical* is the hours dedicated to clinical practice.

We used STATA v14 and Microsoft Excel as statistical software.

### Adequacy of dosage and duration of prescription of antibiotics

We analyzed adequate dosage of antibiotics based on several clinical Guidelines defined in Table 3 [20–25]. This analysis consisted in categorizing prescriptions in those that were under the recommended dose, over the recommended dose, and adequate dose. We also analyzed the number of prescriptions that met the recommended amount of days of treatment. We considered 7 to 10 days as the adequate duration of treatment for all antibiotics included, except for Azithromycin and Penicillin benzathine. We considered 5 days as an adequate duration of treatment for Azithromycin and a single dose for Benzathine penicillin. To define the duration of treatment we used the same clinical guidelines used for the definition of adequate dosage. This analysis was done for each prescription in the patients included in this study.

**Table 3:** Antibiotic Regimens Recommended for URTI

Antibiotic	Dose	Reference
Amoxicillin	*Pediatric:* 50 mg/kg once daily or 25 mg/kg twice daily, High dose 80-90 mg/kg in 2 divided doses (acute otitis media/acute bacterial sinusitis) *Adult:* 500 mg – 2000 mg twice daily	[20, 21]
Cephalexin	*Pediatric:* 20 mg/kg/dose twice daily* Adult:500 mg twice daily	[20, 21]
Benzathine Penicillin G	*Pediatric:* < 27 kg: 600000 U; ≥27 kg: 1200000 U single dose *Adult:* 1200000 U single dose	[20, 21]
Clarithromycin	*Pediatric:* 7.5 mg/kg/dose twice daily *Adult:* 500 mg twice daily	[20, 21]
Azithromycin	*Pediatric:* 12 mg/kg once daily *Adult:* 500 mg daily	[20, 21]
Erythromycin	*Pediatric:* 50 mg/Kg divided four times *Adult:* 500 mg	[24, 25]

## Results

The general characteristics of our sample are described in Table 4.

**Table 4:** Characteristics of the sample

Prescriber	*N* (%)
Total of prescribers	21 (100%)
Age	
Mean (range)	55 (24–65)
Gender	
Female	14 (66.7%)
Male	7 (33.3%)
Classification of Health Professionals	
Rural Trainees	4 (19%)
General Practitioners	7 (33.3%)
Family medicine Doctors	6 (28.6%)
Pediatricians	3 (14.3%)
Psychiatrist	1 (4.8%)
Patients	*N* (%)
Total of patients	1393 (100%)
Age	
Mean (range)	16 (0–93)
Age Groups	
0–17 years	913 (65.5%)
≥18 years	480 (34.5%)
Gender	
Female	804 (57.7%)
Male	589 (42.3%)
Antibiotic prescription	
0–17 years	
Yes	337 (36.9%)
No	576 (63.1%)
≥18 years	
Yes No	186 (38.75%) 294 (61.25%)

### Adequacy and appropriateness of antibiotic prescription

We included 1393 patients that met our inclusion criteria in the analysis that were attended by 21 health professionals (4 Rural Trainees, 7 General Practitioners, 6 Family Medicine Doctors, 3 Pediatricians and 1 Psychiatrist). Out of the 1393 patients, 523 were prescribed antibiotics, constituting an antibiotic prescription rate of 37.5%. Of these 523 patients, 51 required an antibiotic, resulting in an appropriate antibiotic prescription rate of 9.75%. The 51 patients in our sample that needed antibiotics represent 3.66% of all patients. When analyzing each individual case, 33 of these 51 patients received an antibiotic, constituting an adequate prescription rate of 64.7%. We then debriefed the data by different diagnosis according to the ICD-10 codes (Table 5). Acute nasopharyngitis (J00), also known as common cold, represents 41.73% of all diagnoses. Only 2.41% of the cases of common cold had other criteria requiring antibiotics and 2.91% of the cases received antibiotics. Nevertheless, of the cases of J00 that needed antibiotics, only 5.88% received them, the rest of antibiotics prescribed were for cases where it was not needed. This scenario is very different when analyzing prescription for J02, J03 or J06. The results for J02 show us that 51.3% of patients with this diagnosis were prescribed antibiotics, when only 2.94% needed them. The case for acute tonsillitis (J03) is that 83.13% of the cases were prescribed antibiotics, when 9.64% needed them. For Acute upper respiratory infections of multiple and unspecified sites (J06) 61.5% of cases were prescribed antibiotics, when none of them needed them. In the case of suppurative and nonspecified otitis media (H66), all of them needed antibiotics and were all accurately prescribed.

**Table 5:** Appropriateness and adequacy of antibiotic prescription according to ICD-10 diagnosis code

ICD-10 Code	# of patients	Prescribed Antibiotic	Cases that needed antibiotic	Cases that needed + received antibiotics	Appropriate prescription rate (%)	Adequate prescription rate (%)
J00	581	17	14	1	82.36	7.14
J01	93	70	4	4	5.71	100
J02	306	157	9	5	3.18	2.94
J03	166	138	16	15	11.59	93.75
J04	34	12	0	0	–	–
J05	8	0	0	0	–	–
J06	191	117	0	0	0	0
J10	0	0	0	0	–	–
J11	0	0	0	0	–	–
H65	8	6	2	2	33.33	100
H66	6	6	6	6	100	100
Total	1393	523	51	33	9.75	64.70

Appropriate and adequate prescription for codes J04, J05, J10, J11 were not estimated. J00 Acute nasopharyngitis [common cold], J01 Acute sinusitis*,* J02 Acute pharyngitis*,* J03 Acute tonsillitis*,* J04 Acute laryngitis and tracheitis*,* J05 Acute obstructive laryngitis [croup] and epiglottitis, J06 Acute upper respiratory infections of multiple and unspecified sites*,* J10 Influenza due to other identified influenza virus, J11 Influenza due to unidentified influenza virus

Our data includes a classification of all health professionals working at the health center that can prescribe antibiotics. These categories are defined as: General Practitioners, Rural Medical Trainees, Family Medicine Doctors, Pediatricians and Psychiatrists. The results for the analysis of antibiotic prescription depending on the classification of health professionals are shown in Table 6.

**Table 6:** Appropriateness and adequacy of antibiotic prescription according to health professionals

	# of patients	Prescribed Antibiotic	Cases that needed antibiotic	Cases that needed + received antibiotics	Appropriate prescription rate (%)	Adequate prescription rate (%)
General Practitioners (*n* = 7)	692	226	21	14	9.29	66.66
Family Medicine Doctors (*n* = 6)	128	29	9	5	31.03	55.55
Rural Medical Trainees (*n* = 4)	30	17	2	2	11.76	100
Psychiatrists (*n* = 1)	23	23	1	1	4.34	100
Pediatricians (*n* = 3)	520	228	18	11	7.89	61.11
Total	1393	523	51	33	9.75	64.7

Our final multiple logistic regression model included antibiotic prescription as a dependent variable and classification of health professional, sex of prescriber and hours of clinical practice of the health professional as independent variables. We found a statistically significant difference in antibiotic prescription by classification of health professional. We used Family Medicine Doctors as the category of reference for the variable of categories of health professionals. When holding gender of prescriber and hours of clinical practice constant, the odds of antibiotic prescription compare to Family Medicine Doctors was of 4.62 times for Rural Medicine Trainees, 2.58 times for Pediatricians and 1.78 times for General Practitioners. When adjusting for classification of health professional and hours of clinical practice, there was no statistically significant difference in prescription between male and female health professionals (Table 7).

**Table 7:** Odds Ratios of antibiotic prescription

Variable	Odds Ratio (95% CI)	*P*-value
Category of Health Professional		
Family Medicine Doctor (REF)	1.00	
Pediatrician	2.59 (1.64–4.08)	0.000
General Practitioner	1.78 (1.14–2.78)	0.012
Rural Trainee	4.62 (2.00–10.67)	0.000
Gender of Prescriber		
Male (REF)	1.00	
Female	0.89 (0.68–1.18)	0.422
Hours dedicated to clinical practice per day		
0 (REF)	1.00	
1+	1.38 (1.08–1.76)	0.011

REF: Reference category

### Adequacy of dosage and duration of prescription of antibiotics

**Fig. 1:**
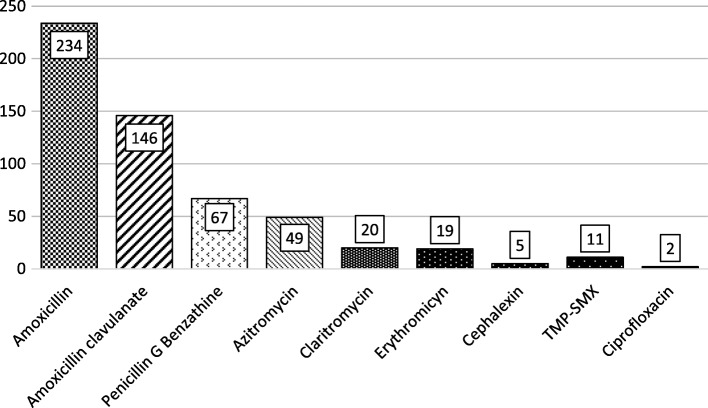
Distribution of types of antibiotics used. Legend: TMP-SMX: Trimethoprim-sulphamethoxazole

Without regarding appropriateness or adequacy of prescription, we analyzed adequacy of dosage of all antibiotic prescriptions. The average number of days of treatment prescribed was of 7.26 days (range 1–15 days) with a mode of 7 days. The distribution of antibiotic classes used are shown in Fig. 1.

Out of the 523 patients that received antibiotics, 493 (94.3%) were prescribed one antibiotic and 30 (5.7%) were prescribed a combination of antibiotics. The two types of combinations prescribed were ‘Benzathine penicillin + Amoxicillin’ in 26 patients (86.7%) and ‘Benzathine penicillin + Azitromycine’ for 4 patients (13.3%). Neither one of the combinations of antibiotics used can be considered as appropriate prescription.

Out of the 337 children that were prescribed antibiotics, 7 received a combination of antibiotics, resulting in 344 antibiotics prescribed in children (single or in combination). Considering weight was reported for all the children included in this study, we were able to calculate the adequate dose for treatment in this group of patients.

**Fig. 2:**
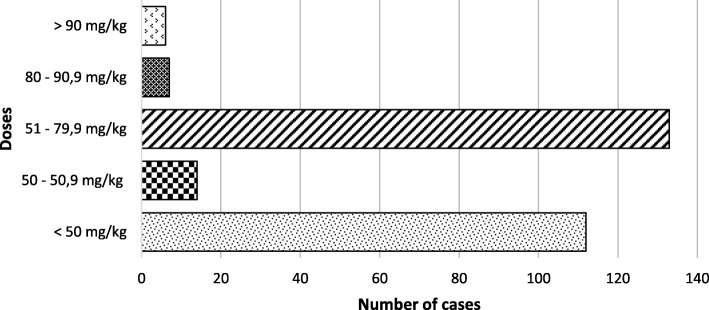
Amoxicillin used doses in children. Legend: *Doses of 50–50.9 mg/kg/day and 80–90.9 mg/kg/day are considered as adequate according to different diagnosis*

Amoxicillin was prescribed in 272 (79.06%) times in children (Fig. 2), of which 6.99% were prescribed with an adequate dose. Out of all the antibiotics prescribed, 16.86% were prescribed with an adequate dose, 48.26% did not reach the adequate dose and 34.88% went over the adequate dose. The description of adequate doses of each antibiotic prescribed for children can be found in Table 8 with further details in Fig. 2.

**Table 8:** Appropriateness of antibiotic dose

Antibiotic	Adequate dose	Under adequate dose	Over adequate dose	Total
Pediatric				
Amoxicillin (otitis and sinusitis)	5 (9.09%)	44 (80%)	6 (10.90%)	55
Amoxicillin (other diagnostics)	14 (6.45%)	112 (51.61%)	91 (41.93%)	217
Azithromycin	2 (18.18%)	6 (54.54%)	3 (27.27%)	11
Clarithromycin	6 (30%)	2 (10%)	12 (60%)	20
Erythromycin	15 (83.33%)	2 (11.11%)	1 (5.55%)	18
Penicillin G Benzathine	12 (70.59%)	0 (0%)	5 (29.41%)	17
Cephalexine	2 (50%)	0 (0%)	2 (50%)	4
Trimethoprim-sulphamethoxazole	2 (100%)	0 (0%)	0 (0%)	2
Total	58 (16.86%)	166 (48.26%)	120 (34.88%)	344
Adults				
Amoxicillin (otitis and sinusitis)	20 (100%)	0 (0%)	0 (0%)	20
Amoxicillin (other diagnostics)	85 (100%)	0 (0%)	0 (0%)	85
Azithromycin	37 (97.36%)	0 (0%)	1 (2.63%)	38
Clarithromycin	3 (100%)	0 (0%)	0 (0%)	3
Erythromycin	1 (100%)	0 (0%)	0 (0%)	1
Penicillin G Benzathine	38 (76%)	0 (0%)	12 (24%)	50
Cephalexine	1 (100%)	0 (0%)	0 (0%)	1
Trimethoprim-sulphamethoxazole	9 (100%)	0 (0%)	0 (0%)	9
Ciprofloxacin	1 (50%)	0 (0%)	1 (50%)	2
Total	195 (93.30%)	0 (0%)	14 (6.70%)	209

Out of the 186 adults that were prescribed antibiotics, 23 received a combination of antibiotics, resulting in 209 antibiotics prescribed in adults. Amoxicillin was prescribed 105 times in adults, all of which were prescribed with an adequate dose. Out of all antibiotics prescribed in adults, 93.30% were prescribed with an adequate dose, 0% did not reach the adequate dose and 6.70% went over the adequate dose. The description for the adequate dose of each antibiotic prescribed for adults can be found in Table 8 with further details in Fig. 2.

We then analyzed the number of prescriptions that met the recommended duration of treatment. Out of all the prescriptions, 85.66% met the recommended duration of antibiotic treatment, 9.37% were prescribed less than the recommended duration and 4.97% went over the recommended duration (Table 9).

**Table 9:** Duration of Prescription of Antibiotics

Antibiotic	Meets recommended duration of treatment	Under recommended duration of treatment	Over recommended duration of treatment	Total
All antibiotics prescribed that have a recommendation of 7–10 days of treatment	388 (88.79%)	24 (5.49%)	25 (5.72%)	437
Azithromycin (Recommendation of 5 days of treatment)	23 (46.94%)	25 (51.02%)	1 (2.04%)	49
Penicillin G Benzathine (Recommendation of one single dose)	37 (100%)	0 (0%)	0 (0%)	37
Total	448 (85.66%)	49 (9.37%)	26 (4.97%)	523

## Discussion

The results of our study report a 90.25% of inappropriate prescription in the Health Center that we evaluated. An estimated average of antibiotic prescription appropriateness from studies around the world, mostly including high – middle income countries, is of about 50% [8, 26–31]. A review of 344 studies between 1990 and 2009 on the treatment of childhood infections in 78 low-middle income countries reported that a high percentage of viral upper respiratory tract infection cases were being treated with antibiotics, with this percentage increasing over time (from 42% before 1990 up to 72% in 2006–2009) [32]. This same study reports 47.1% of inappropriate antibiotic use in URTI in lower-middle income countries and 25.8% for Latin America.

This means that our study reflects almost one eighth of appropriate prescription as compared to other countries around the world. This astonishing number allows us to realize the amount of work that needs to be done locally to improve this rate.

Studies analyzing the same issue also evaluated the rate of antibiotic prescription and compared their results to a baseline. These studies did not consider if those prescriptions were necessary or not or if the dosage of antibiotics was adequate, but they did calculate the rate of antibiotic prescription that we can compare our study to. Cordoba, et al. [11] published a study in 2016 that evaluated antibiotic prescription in patients with a suspected diagnosis of URTI in primary care health centers in four Latin-American countries. The reported antibiotic prescription rate was of 35% in Argentina, 40% in Bolivia, 24% in Paraguay and 27% in Uruguay. Another study by Doubova et al. [33] in Mexico reported more than 61% of children diagnosed with non-streptococcal URTI received antibiotics after the first visit to the health facility. These studies did not evaluate the appropriate prescription rate but show that in a comparable context the rate of antibiotic prescriptions is very similar.

A study about Denmark and Iceland published in 2015 [34] evaluated antibiotic prescription in URTI by general practitioners. The results demonstrate a prescription rate of 59.3% in Denmark and 75.8% in Iceland. Appropriate antibiotic prescription was also assessed by analyzing days of symptoms and Centor Criteria, possibly underestimating inappropriate use. Their results show a rate of 16.4% for sinusitis and 26.3% for pharyngitis in Denmark and 17.8 and 21.3% in Iceland. Another study done in 6 countries [35] (Switzerland, Denmark, Argentina, Spain, Russia and Lithuania) reported a 50% inappropriate prescription rate and close to 100% inappropriate prescription for rhinopharyngitis and otitis media. These studies show very low appropriate antibiotic prescription rates like the one we found in our study.

It is as important to note the cases of inadequate care, where patients needed antibiotics and did not receive them. Of the total number of cases that required antibiotic prescription, 35.2% did not receive antibiotics. Considering the 93.7% of over-prescription, we would recommend further studies to identify the cause of this rate.

When we analyze the data by diagnosis, we see that the antibiotic prescription rate for common cold is very low, as it should be according to the clinical guideline used to describe appropriateness of prescription for this study. This allows us to see that the common cold is not a major problem of antibiotic prescription in this case. The data shows that with any diagnosis different from a common cold, health professionals at this health center tend to prescribe antibiotics at a much higher rate. There is a particular concern with “acute upper respiratory infections of multiple and unspecified sites” (J06), where the group of identified symptoms can reflect other diagnosis, but are classified in this category that leaves an open diagnosis. In this case, patients received antibiotics at a high rate, where it is not appropriate in any case. It seems that the vague definition of this category allows for a lot of variation in treatment prescribed.

In Ecuador, all Medical graduates must do a year of training in a rural area in order to be able to practice as a General Practitioner. In North America and Europe, the General Practitioner is being replaced by Family Medicine Doctors, but in Ecuador the General Practitioner is still the most common type of doctor. In our study, when classifying by health professions, Family Medicine Doctors have the lowest odds of prescribing antibiotics. This could be due to the additional years of training that are required to fulfill that degree, giving them the necessary tools to act. The clinical guideline used in this study does not take into consideration psychiatric comorbidities as a parameter for prescription. Even though psychiatric patients tend to have associated comorbidities that are not considered in this study, patients in primary care health centers usually do not have severe psychiatric disorders that could justify an antibiotic prescription due uniquely to their psychiatric condition.

A study by Del Fiol et al. [36], that analyzes the use of antibiotics in children in two Health Centers in Brazil, reports that 50% of the prescribed dosages of amoxicillin are under the recommended amounts according to Brazilian guidelines. These results are similar to those in our study that show 57.35% of prescriptions were under the recommended dose for the same antibiotic in children.

One of the most relevant limitations in our study is the fact that we relied on retrospective information from EHR. The EHR used in this district allows for registration of all possible variables, but health professionals in many occasions fill in only what is relevant to them about the current reason of consultation, leading to possible incomplete records. Even though during the consultation the health professional may not include the patients’ medical history, like history of prematurity, previous hospitalization and some comorbidities, they are able to find this information in the records and can refer to them in order to decide on diagnosis and treatment. This thought process is not contemplated in our analysis, turning it into a limitation for our study and a possible underestimation of appropriate prescription in some cases. Nevertheless, all the variables described in the clinical guidelines were both registered in all patients included and considered for analysis.

Another limitation is the lack of a National Clinical Guideline for upper respiratory tract infections, forcing us to use the NICE guideline. NICE Guidelines consider a global context, instead of only local studies, for their recommendations, which is why we considered this guideline to be appropriate for our study. Ecuador’s Minister of Health recommends the use of international guidelines when there is no local guideline provided. NICE guidelines are always taken into consideration when developing national guidelines in Ecuador, which is why we chose this as our reference.

Another limitation to our study is that the sample comes from only one health center. This is due to the lack of an EHR that can report the included variables for each patient, which led to having to extract all the data manually.

Finding a difference both between health professionals in the amount of antibiotics each prescribe can be helpful when targeting individuals for educational plans about antibiotics. If these variables were analyzed in each health center, the specific health professionals that need educational interventions about the appropriate use of antibiotics can be targeted.

## Conclusion

In the health center in Ecuador included in the study there is a high rate of inappropriate prescribing of antibiotics; these findings support the need for implementation of strategies to reduce the prescription of antibiotics. Creating this baseline for the whole country instead of one health center could be the first step to realizing the scope of the problem in Ecuador. The antibiotic prescription, appropriate prescription and adequate prescription rates all underscore the need for further research and specific policy analysis and implementation in developing countries. Knowing that inadequate antibiotic prescribing is a problem in this local setting can contribute to an appropriate prescription policy.

## Additional files

Additional file 1: “Description of Variables”, shows variables collected for the analysis of appropriateness and adequacy of antibiotic prescription. (DOCX 23 kb)

Additional file 2: “Variables considered for the regression model”, shows variables chosen for bivariate logistic regression model. (DOCX 21 kb)

## Abbreviations

EHR: Electronic Health Records
ICD: International Classification of Disease
NICE: National Institute for Health and Care Excellence
URTI: Upper Respiratory Tract Infection
WHO: World Health Organization
