# Supplementary material for: “Appropriateness and adequacy of antibiotic prescription for upper respiratory tract infections in ambulatory health care centers in Ecuador”
Source: BMC Pharmacol Toxicol. 2018 Jul 27;19:46. doi: 10.1186/s40360-018-0237-y (PMC6062893; doi:10.1186/s40360-018-0237-y)
Supplement: Supplementary file 1 — “Description of Variables”, shows variables collected for the analysis of appropriateness and adequacy of antibiotic prescription. (DOCX 23 kb) [file 40360_2018_237_MOESM1_ESM.docx]

**Additional file 1**

**Description of Variables**

| **Variable** | **Definition** | **Detail** | **Scale** |
| --- | --- | --- | --- |
| **Gender of prescriber** | Gender of identification of the health professional |  | (0): Male  (1):Female |
| **Age of prescriber** | Age of prescriber in years | 18 – 39 years old  40 – 64 years old  ≥65 years old | Age in years |
| **Highest level of education** | Highest degree of health education | Health professional in “rural year” of training  Health professional defined as general practitioner  Health professional in a medical residency program  Pediatrician  Health professional with Family Medicine Training | (1) Rural Trainee  (2) General Practitioner  (3) Medical Resident  (4) Pediatrician  (5) Family medicine |
| **Time working in same health center** | Time working in the health center | Number of years health professional has worked in a public Health Center according to Human Resources. | Time in years |
| **Hours dedicated to clinical practice** | Time dedicated to clinical practice by the health professional | Number of hours health professional assists clinical practice in Health Center | Time in hours |
| **Patient age** | Age of patient in years | <2 years  2 – 4 years  5 – 9 years  10 – 17 years  18 – 39 years  40 – 64 years  ≥65 years | Age in years |
| **Patient sex** | Gender of identification of patient |  | (0): Male  (1): Female |
| **Code of diagnosis** | Diagnosis of patient according to ICD-10 codes | J00  J01  J02  J03  J04  J05  J06  J10  J11  H65  H66 | (1) Acute Nasopharyngitis (common cold)  (2) Acute Sinusitis  (3) Acute pharyngitis  (4) Acute Tonsillitis  (5) Acute laryngitis and tracheitis  (6) Acute obstructive laryngitis (crup) and epiglottitis  (7) Acute upper respiratory infections of multiple and unspecified sites  (8) Influenza due to other identified influenza virus  (9) Influenza due to unidentified influenza virus  (10) Nonsuppurative otitis media  (11) Suppurative and non-specified otitis media |
| **Comorbidity** | Medical condition that that exists simultaneously but independent of another condition in the patient.  Comorbidity:  *Cardiac*  *Pulmonary*  *Renal*  *Hepatic*  *Neuromuscular*  *Immunosuppression*  *Cystic fibrosis* | Presence of 1 or more comorbidities  No comorbidities | (1) With comorbidities  (2) Without comorbidities |
| **Diabetes Mellitus** | Diabetes mellitus | Personal history of Diabetes mellitus | (1) Yes  (2) No |
| **Use of corticosteroids** | Current use of corticosteroids |  | (1) Yes  (2) No |
| **Child with history of prematurity** | Personal history of prematurity in children <2 years old. | Born at less than 37 weeks | (1) Yes  (2) No |
| **History of Hospitalization** | Personal history of hospitalization | History of hospitalization in past year | (1) Yes  (2) No |
| **Antibiotic prescription** | Act of prescribing antibiotics to a patient | Record of prescription associated to every consult | (1) Yes  (2) No |
