# Supplementary material for: “Appropriateness and adequacy of antibiotic prescription for upper respiratory tract infections in ambulatory health care centers in Ecuador”
Source: BMC Pharmacol Toxicol. 2018 Jul 27;19:46. doi: 10.1186/s40360-018-0237-y (PMC6062893; doi:10.1186/s40360-018-0237-y)
Supplement: Supplementary file 2 — “Variables considered for the regression model”, shows variables chosen for bivariate logistic regression model. (DOCX 21 kb) [file 40360_2018_237_MOESM2_ESM.docx]

**Additional file 2**

**Variables considered for the regression models**

| **Variable** | **Definition** | **Detail** | **Scale** | **Type of variable** |
| --- | --- | --- | --- | --- |
| **Antibiotic prescription** | Act of prescribing antibiotics to a patient | Record of prescription associated to every consult | (1) Yes  (2) No | Dichotomous |
| **Gender of prescriber** | Gender of identification of the health professional |  | (0): Male  (1):Female | Dichotomous |
| **Age of prescriber** | Age of prescriber in years |  | Age in years | Continuous |
| **Classification of Health Professionals** | Highest degree of health education | Health professional with Family Medicine Training  Pediatrician  Health professional defined as general practitioner  Health professional in “rural year” of training | (0) Family medicine  (1) Pediatrician  (2) General Practitioner  (3) Rural Trainee | Categorical |
| **Time working in same health center** | Time working in the health center | Number of years health professional has worked in a public Health Center according to Human Resources. | Time in years | Continuous |
| **Hours dedicated to clinical practice** | Time dedicated to clinical practice by the health professional per day | Number of hours health professional assists clinical practice in Health Center | Time in hours | Continuous |
| **Patient age** | Age of patient in years |  | Age in years | Continuous |
| **Patient sex** | Gender of identification of patient |  | (0): Male  (1): Female | Dichotomous |
